# Microbubble-enhanced dielectric barrier discharge pretreatment of microcrystalline cellulose

**DOI:** 10.1016/j.biombioe.2018.08.005

**Published:** 2018-11

**Authors:** Alexander Wright, Adam Marsh, Federica Ricciotti, Alex Shaw, Felipe Iza, Richard Holdich, Hemaka Bandulasena

**Affiliations:** a Department of Chemical Engineering, Loughborough University, Loughborough, Leicestershire, LE11 3TU, United Kingdom; b Department of Civil, Chemical, Environmental and Materials Engineering, University of Bologna, 40136, Italy; c Wolfson School of Mechanical, Electrical and Manufacturing Engineering, Loughborough University, Loughborough, Leicestershire, LE11 3TU, United Kingdom

**Keywords:** Micro crystalline cellulose, DBD plasma, Ozone, Microbubbles, Enzymatic hydrolysis

## Abstract

Cellulose recalcitrance is one of the major barriers in converting renewable biomass to biofuels or useful chemicals. A pretreatment reactor that forms a dielectric barrier discharge plasma at the gas-liquid interface of the microbubbles has been developed and tested to pretreat α-cellulose. Modulation of the plasma discharge provided control over the mixture of species generated, and the reactive oxygen species (mainly ozone) were found to be more effective in breaking-up the cellulose structure compared to that of the reactive nitrogen species. The effectiveness of pretreatment under different conditions was determined by measuring both the solubility of treated samples in sodium hydroxide and conversion of cellulose to glucose via enzymatic hydrolysis. Solutions pretreated under pH 3 buffer solutions achieved the best result raising the solubility from 17% to 70% and improving the glucose conversion from 24% to 51%. Under the best conditions, plasma-microbubble treatment caused pronounced crevices on the cellulose surface enhancing access to the reactive species for further breakdown of the structure and to enzymes for saccharification.

## Highlights

• Plasma-microbubble pretreatment at pH 3 increased cellulose solubility from 17% to 70%.
• Pretreatment aided cleavage of hydrogen bonds linking the polymeric structure.
• Pronounced crevices were observed at the cellulose surface following pretreatment.
• Limited accessibility to the crystalline cellulose is a barrier to pretreatment.

## Graphical abstract

**Figure:**
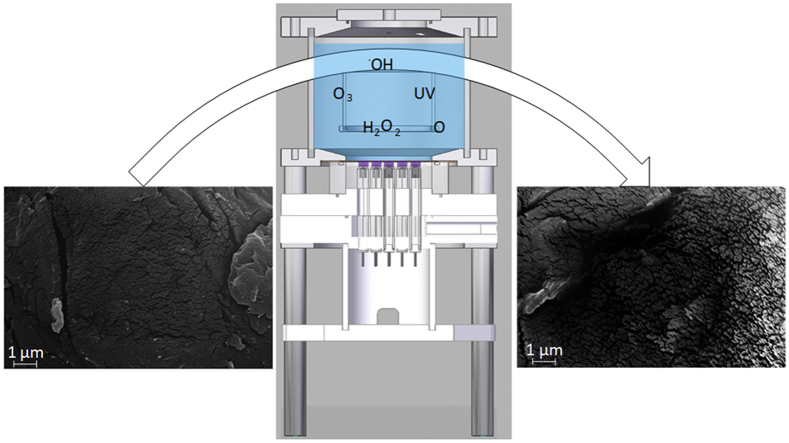

## 1 Introduction

Depletion of fossil fuels and emission of greenhouse gases have led to an increase in demand for sustainable biofuels derived from renewable sources [1,2]. Biofuels can be broadly categorised as first generation (mainly sugars and starch), second generation (lignocellulosic biomass) and third generation (algae). Biofuels derived from lignocellulosic materials have gained significant attention recently due to criticism over first generation biofuels for using resources allocated for food production, needing government subsidies to be competitive and high net greenhouse gas emissions. In lignocellulosic biomass, approximately two-thirds of the total dry weight consist of cellulose and hemicellulose, connected either by covalent or hydrogen bonds and shielded by lignin. Bioethanol or biogas is mainly derived from cellulose and hemicellulose [3,4], while lignin can be combusted to generate heat and electricity. Pure cellulose can be isolated from feedstocks such as lignocellulosic biomass, cotton and bacterial cellulose using highly commercialised pretreatment methods that often include multiple stages of treatments [5]. α-cellulose isolated from such treatment methods contains both amorphous and crystalline parts, and through further treatment, the amorphous sections can be broken-down to form microcrystalline cellulose (MCC). However, extensive treatments significantly increases the cost MCC production; hence their use is often limited to pharmaceutical, cosmetic and food industries [5].

Cellulose is often hydrolysed by enzymes or acids to form glucose, which can then be fermented by yeast to produce bioethanol and other chemical by-products [1]. Alternatively, cellulose can also be converted into biogas by anaerobic digestion. In either case, the conversion rate is often limited by crystallinity of cellulose making the polymer hard to degrade as well as being insoluble in most solvents [6,7]. There is significant interest in increasing the solubility of cellulose for improving hydrolysis or facilitating accessibility by anaerobic bacteria [7]. Steam explosion [8] and chemical treatment [9,10] are commercially developed pretreatment methods to achieve this task, but combination of several methods have proven to be more effective [11,12]. However, a large proportion of the costs associated with biofuel production from lignocellulosic biomass is spent on pretreatment; therefore, cheap and more effective methods are sought to make the process viable.

Recently, atmospheric pressure plasmas (APPs) have received increased attention due to its ability to produce a wide range of highly reactive species that can potentially break-up the highly crystalline cellulose structure. When air is used as the feed gas, APPs produce reactive oxygen species (ROS) such as ozone, hydrogen peroxide and hydroxy radicals and reactive nitrogen species (RNS) such as nitric acids and peroxynitrites [13]. The concentration of each reactive species in the cocktail of gaseous output depends on the conditions used to produce the plasma such as electrical characteristics of the power supply and composition of the feed gas. The use of APPs to reduce crystallinity of cellulose has been reported by Jun et al., where an argon feed jet was used to treat cotton fibre with an α-cellulose content of 96.8% [14]. Both, increased treatment time and power applied to the plasma improved the solubility of cellulose. A decrease in hydrogen bonds was observed within the cellulose structure as solubility increased. It should be noted that, whilst high solubility is achievable through the use of APPs, ozone can also damage the cellulose structure which selectively attacks C=C through ozonolysis [15].

Plasma jets can produce high concentrations of reactive species within a small area of interest, but their scalability is limited due to small volumes of liquid treated by each jet. There is significant interest in treating large volumes at industrial scale using APPs; hence scalable designs and technologies are needed. Huang et al., designed a pretreatment reactor that produces a Dielectric Barrier Discharge (DBD) plasma above the liquid surface to treat MCC solutions [16]. It was found that the carrier gas had no significant effect on the pretreatment, and the radical species generated by the plasma such as hydroxyl radicals, hydrogen peroxide and hydrated electrons were mainly responsible for the depolymerisation effectiveness. A 50-min pretreatment of MCC in this reactor reduced the crystallinity index (CI) from 82.8% to 58.9%, demonstrating the suitability of plasma treatment for reducing intramolecular and intermolecular bonds. Even though this study demonstrated that radicals and ROS were the key species for reducing crystallinity of MCC, the effect of RNS should not be neglected. In a separate study, NO_2_ has been shown to effectively oxidise cellulose to glucose, reducing the need for a further processing either by enzymes or acid hydrolysis [17].

The conversion of MCC into glucose without either acid or enzymatic hydrolysis has also been facilitated by plasma. Prasertsung at al. used two opposing submerged electrodes to hydrolyse cellulose into reducing sugars directly [18]. The iron particles sputtered into the solution from the iron electrodes reacted with hydrogen peroxide to form high concentrations of hydroxy radicals through Fenton reactions. Hydroxy radicals are known to breakdown complex molecules effectively; however they often react indiscriminately and can break down sugars [19]. Whilst this approach sounds promising in reducing the costs associated with enzymatic hydrolysis or acid treatments in producing sugars, the issue of scalability and additional cost of removing nanoparticles from the liquid remains. Therefore, this study will focus on facilitating the hydrolysis process of α-cellulose with reactive species generated from a DBD plasma and identifying the limiting factors that may be present.

Energy efficient fluidic oscillation-mediated microbubbles have been demonstrated to improve mass transfer rates significantly due to their high surface to volume ratio, and this technology has been applied to various processes needing efficient gas-liquid mass transfer such as waste water treatment, aqua culture and bioreactors [20,21]. In addition to improved mass transfer, microbubbles provide efficient mixing of the reactor contents, removing the need for mechanical mixing; hence reducing the energy requirement for pretreatment [22]. This plasma-microbubble reactor treatment has been demonstrated as an effective approach in pretreating lignocellulosic biomass for bioethanol production [23].

The main purpose of this study is to determine the effectiveness of plasma-microbubble pretreatment for improving solubility and digestibility of cellulose. Pure α-cellulose was chosen as the feedstock over raw biomass to isolate the effect of reactive species produced by the APP on cellulose fraction of the biomass. Optimum operating conditions for the reactor have been determined, and a pretreatment procedure has been developed to maximize the effect. This paper is organised as follows. In Section 2, plasma-microbubble reactor used in this study and various characterisation tests are described under Materials and Methods. In Section 3, results on the reactor performance and effectiveness of the pretreatment method used is discussed. In section 4, conclusions are drawn.

## 2 Materials and methods

### 2.1 Plasma-microbubble reactor

**Fig. 1:**
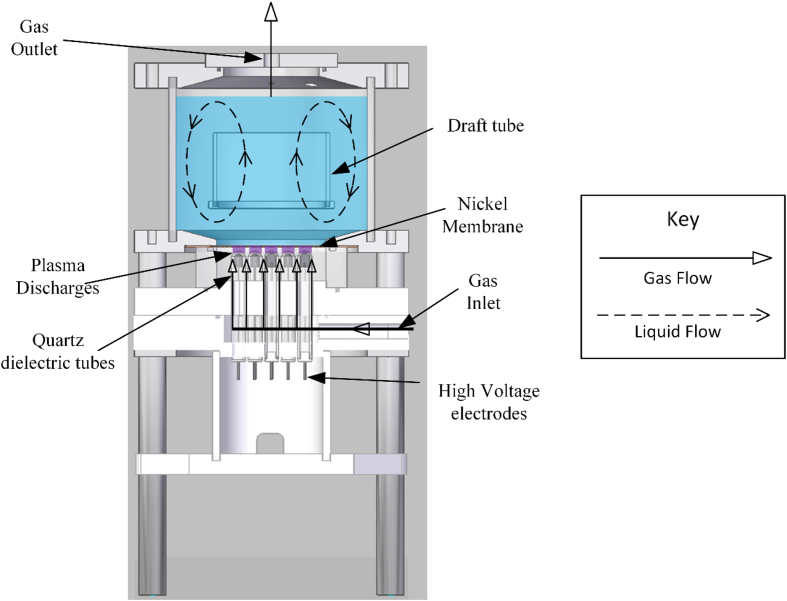
A schematic diagram of the plasma-microbubble reactor.

The reactor used in this study is similar to that described in Ref. [23], forming a DBD plasma at the gas-liquid interface and dispersing the reactive species generated using microbubbles. The microporous nickel membrane used for microbubble generation also acts as the ground electrode, making it possible to generate the plasma in situ and at the gas-liquid interface. Generation of plasma at the microbubble generation site allows immediate transfer of short-lived species into the liquid phase in addition to the long-lived ROS and RNS. A schematic diagram of the modified reactor configuration is shown in Fig. 1. The reactor mainly consists of two parts: the plasma module and the reaction tank. The plasma module consists of 19 stainless steel rods as the high voltage electrode and a microporous nickel membrane as the ground electrode. Each stainless-steel rod is shielded by a 1.5 mm thick quartz crucible, which acts as the dielectric barrier. The gap between the high voltage electrode and the ground electrode is set to 2 mm in this study. The feed gas is supplied at 1.25 SLPM via a mass flow controller (MKS, PR4000B). The inlet gas then flows around the quartz crucibles before reaching the plenum chamber, where plasma is formed. The gas flow through the porous membrane produces ozone rich microbubbles ∼550 μm inside the reaction tank. The reaction tank has a maximum usable capacity of 1.0 L, and the height and the internal diameter are 100 mm and 140 mm respectively. A draft tube with a height of 50 mm and a diameter of 80 mm is positioned in the tank as shown in Fig. 1. The draft tube dimensions and placement are calculated according to [22].

To form a plasma discharge between the electrodes and to generate reactive species, an in-house built full-bridge resonant power supply operating at a frequency of 29 kHz and a voltage of 10.6 kV_pp_ was used. The plasma discharge was modulated by introducing a duty cycle to the circuitry. The duty cycle was defined as the percentage of time that the electrodes are supplied with a high voltage in each cycle. The desired duty cycle was achieved by changing the off-time while keeping the on-time fixed at 100 ms. The average power draw of the plasma depends on the duty cycle used; hence for 10%, 45% and 60% duty cycles used in this study, 4.2, 19.0 and 25.3 W were drawn respectively.

### 2.2 Gas phase Fourier transform infrared spectrometry (FTIR)

To characterise the effluent of the plasma discharge, gas phase FTIR was used. Here, the exhaust gas from the empty reactor (with no liquid) was fed directly into a gas cell (1–16 m, Pike technologies Ltd) mounted within the FTIR (4700, Jasco Ltd). The ozone concentration was measured at the absorbance band at 2122 cm^−1^, N_2_O at 2240 cm^−1^, N_2_O_5_ at 750 cm^−1^, HNO_3_ at 890 cm^−1^, NO at 1900 cm^−1^ and NO_2_ cm^−1^. The concentrations were then calculated from the beer-lambert law using the cross sections read from the HITRAN data base [24].

### 2.3 Cellulose pretreatment

20 g/L α-cellulose (VWR, UK) suspensions were prepared by mixing dry cellulose powder in water with an overhead stirrer for 1 h prior to pretreatment. Suspensions were made up with either tap water, a pH 3 buffer (0.1 M phosphoric acid/sodium phosphate dihydrate) a pH 7 buffer (0.1 M sodium phosphate monobasic/sodium phosphate dibasic) or a pH 9 borate buffer (0.1 M boric acid/sodium hydroxide). All chemicals were supplied by Fisher Scientific, Ltd., UK. Following the pretreatment, all samples where filtered, washed three times with deionised water and dried in an oven at 50 °C for 24 h prior to analysis.

### 2.4 Solubility analysis

Solubility of α-cellulose in sodium hydroxide has been used as an indicator of the crystallinity of the cellulose structure and can be used to estimate the effectiveness of pretreatment [25]. First, 5 g of dried cellulose sample ($m_{i})$ was added to 96 g of 2.275 M sodium hydroxide solution and mixed for 2 h at 4 °C. Then, the suspension was centrifuged at 5000 g in a temperature-controlled centrifuge (J2-21 M/E, Beckman) for 1 h, maintaining a temperature of 4 °C. The liquid fraction was then removed, and the solids were neutralised with hydriodic acid (0.35 M). Finally, this suspension was filtered, washed and dried. The remaining solids were then weighed $\left(m_{i}\right)$ and the percentage solubility was calculated according to equation (1).

(1) $$S=\left(\frac{m_{i}-m_{f}}{m_{i}}\right)\times 100\%\;$$

where $m_{i}$ is the initial weight of the cellulose sample and $m_{f}$ is the final dried weight of the sample following the above procedure.

### 2.5 Glucose conversion

Pretreated cellulose samples were hydrolysed using enzymes to determine whether high degree of breakdown leads to improved glucose conversion. A 150 ml suspension of acetate buffer (pH 5) containing 5 g of pretreated α-cellulose and 0.9 ml of cellulase from *Trichoderma reesei* enzyme (units/g > 700, Sigma Aldrich, Ltd) was kept in a rotary incubator for 7 days at 50 °C and 150 rpm. The resulting glucose concentration was then measured using a glucose analyser (GL6, Anolox Ltd).

### 2.6 Scanning electron microscopy (SEM)

Pretreated and raw α-cellulose samples were visualised using SEM. SEM images allow inspection of the surface morphology and could provide an insight into how the structure has been affected by the pretreatment. Samples were sputter coated (SC7640, Quorum Technologies Ltd) with gold palladium for 180 s at 25 mA before imaging with SEM (LEO 1530 V P, Carl Zeiss Ltd) using an excitation voltage of 20 KV and an aperture of 30 μm.

### 2.7 Attenuated total reflection Fourier transform infrared spectrometry (ATR-FTIR)

As a further investigation of the pretreated α-cellulose structure, ATR-FTIR analysis was carried out to determine any changes to chemical bonds. Solid samples were analysed by measuring the attenuated total reflection (ATR) with a ATR sample holder (GS10800-X, Quest Ltd) mounted within an infrared spectrometer (IRAffinity-1, Schimadzu) with a resolution of 4 cm^−1^, measuring between 4000 and 800 cm^−1^ and averaging over 64 scans.

## 3 Results and discussion

### 3.1 Duty cycle optimisation

**Fig. 2:**
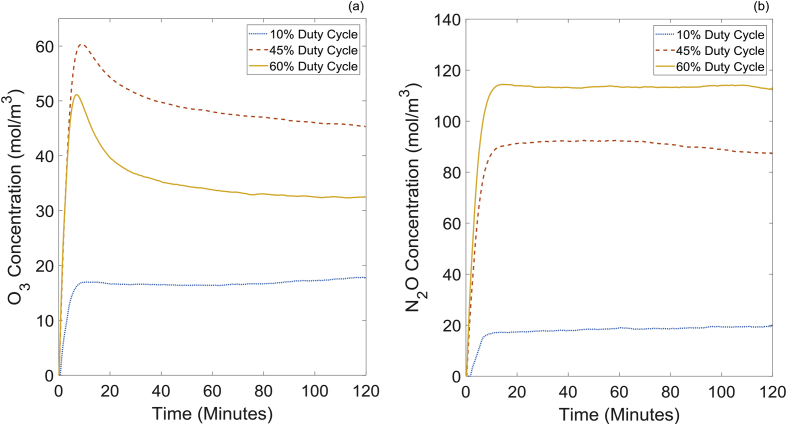
FTIR analysis of (a) O_3_ and (b) N2O at 10%, 45% and 60% duty cycles.

By changing the duty cycle of the reactor, it is possible to manipulate the plasma chemistry; hence produce different concentrations of ROS and RNS. To measure these species in the plasma effluent, FTIR analysis was carried out on 10%, 45% and 60% duty cycles. The concentration of O_3_ and N_2_O in the gas outlet for varying duty cycles is shown in Fig. 2. All concentration profiles reached a steady value after ∼20 min, as there was a slight delay in filling the FTIR column with the plasma effluent. 10% duty cycle produced the lowest concentration of O_3_ and N_2_O, as the input power is the lowest compared to that of the other duty cycles tested. Highest O_3_ and N_2_O concentrations were reported for 45% and 60% duty cycles respectively. For optimum O_3_ production, plasma should be maintained at sufficiently low temperatures by modulating the plasma discharge while providing the maximum energy input. According to Fig. 2 and 45% duty cycle provided the most favourable conditions for O_3_ generation. Low concentrations (<5 moL/m^3^) of RNS such as NO, NO_2_, N_2_O_5_ and HNO_3_ were detected with all duty cycles. However, 60% duty cycle produced the highest concentration of RNS due to high plasma temperatures leading to decomposition of ozone into NO_2_ [26]. High duty cycles of ∼60% have a shorter off-time compared to that of 45% or 10% duty cycles and does not provide sufficient time for the feed gas to cool the plasma module; therefore, heat build-up in the plasma module leads to high RNS.

**Fig. 3:**
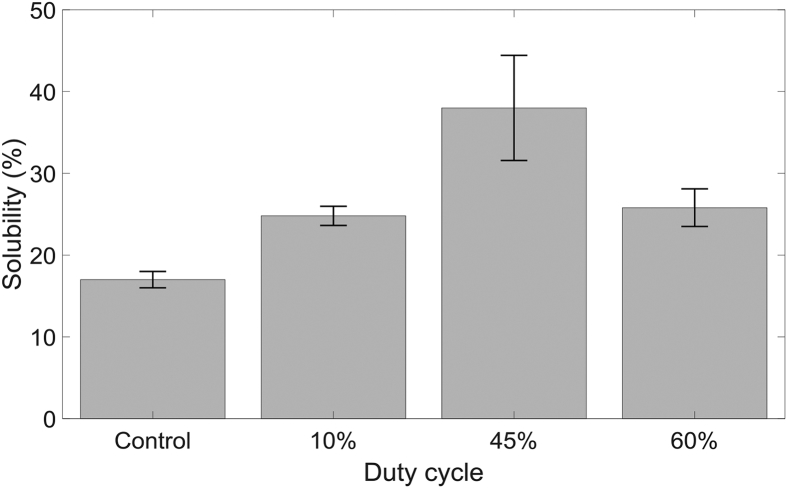
The influence of duty cycle on the solubility of α-cellulose after a 2-h pretreatment. Error bars represent standard error.

To determine the most suitable duty cycle for pretreatment, a suspension containing 20 g/L of α-cellulose was treated in the reactor for 2 h at different duty cycles. Tap water was used for all experiments as preliminary studies showed no significant difference in performance when deionised water was used over tap water. In addition to this, use of deionised water at industrial scale operation will not be feasible. The solubility of the α-cellulose for various duty cycles is shown in Fig. 3.

According to Fig. 3, all pretreated samples showed improved solubility compared to that of the control experiment. However, the maximum solubility was limited to ∼37%, which corresponds to 45% duty cycle operation. Huang et al.,. demonstrated that ROS, especially hydroxy radicals, are responsible for the degree of depolymerisation of α-cellulose compared to that of RNS [16]. Therefore, the maximum solubility observed in this experiment for 45% duty cycle agrees with their results, as the highest O_3_ concentration was reported for the same duty cycle (Fig. 2). Cellulose solubilities achieved for the pretreatment experiments operated at 10% and 60% duty cycles were nearly equal, even though there is a significant difference in ROS and RNS measured in the gas outlet. According to Fig. 2, the average O_3_ concentration for 60% duty cycle is approximately twice that of 10% duty cycle, and the average N_2_O concentration for 60% duty cycle is approximately six times that of 10% duty cycle. This result may suggest that 60% duty cycle should perform better at pretreatment compared to 10% duty cycle as ROS and RNS are much higher for the higher duty cycle. However, once dissolved in the liquid phase, these species will combine with other reactive species to form various products. Measurement of radicals and other reactive species in the liquid phase or at the plasma-liquid interface is extremely challenging due to the short lifetime of these species, the limited means of selectively identifying these species and the lack of optical access in the reactor. Therefore, it is difficult to corelate reactive species in the gas phase to pretreatment effectiveness directly. Since 45% duty cycle provided the highest solubility, it was used throughout the rest of this study.

### 3.2 Effect of pretreatment time

**Fig. 4:**
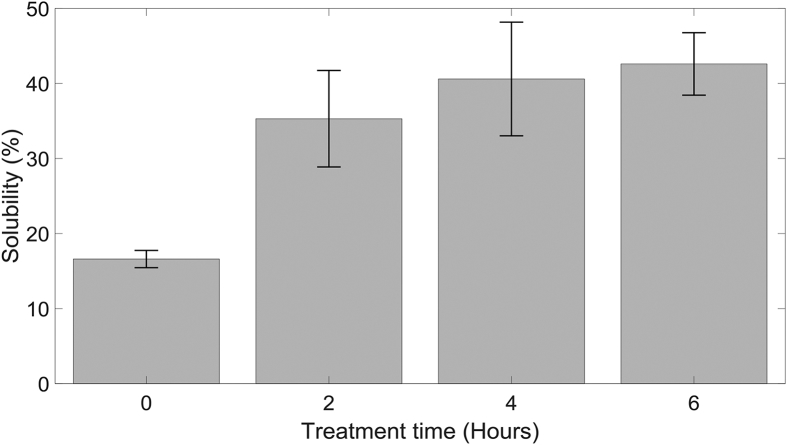
The effect of pretreatment time on solubility at 45% DC. Error bars represent standard error.

As the percentage solubility observed after a 2-h pretreatment was limited to ∼37%, further experiments were carried out to study the effect of pretreatment time on solubility. 20 g/L α-cellulose solutions were treated for 2, 4 and 6 h continuously using a 45% duty cycle. Fig. 4 demonstrates that increased pretreatment time improves solubility, but the values plateau around 6 h of treatment showing a clear limitation. The difference in solubility for 2-h and 6-h pretreatment are marginal and the maximum solubility is limited to ∼45%.

**Fig. 5:**
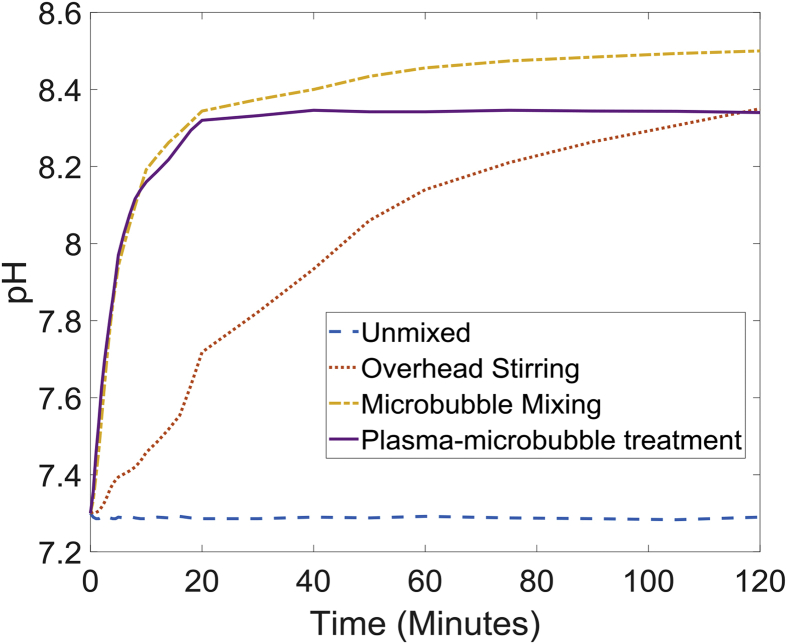
pH change in the bulk liquid (tap water) containing α-cellulose with time for: unmixed reactor, mixed with an overhead stirrer, mixed with microbubble and during pretreatment with plasma-microbubbles.

To investigate solubility limitation, solution pH was measured and analysed for several tests, as water serves as the medium through which the reactive species are transferred from gas plasma to the cellulose surface. A pH probe was placed mid-depth of the reactor in the downcomer region, and measurements were taken every minute for 2 h. Four separate experiments were run for this purpose: unmixed cellulose in the reactor, cellulose mixed with an overhead stirrer, bubbling without plasma and bubbling with plasma module on. Results are shown in Fig. 5. The first observation was that α-cellulose gradually increase solution pH from ∼7.3 to ∼8.4 under mixed conditions (either overhead stirring or using bubbles). However, without any pretreatment, α-cellulose is insoluble in water [5]. A separate experiment was carried out by adding α-cellulose to tap water and deionised (DI) water separately and monitoring pH for 2 h. For the DI water, pH dropped from 6.4 to 5.6 while the pH of tap water increased from 7.3 to 8.34. As the only difference between DI water and tap water is the presence of minerals such as calcium carbonate, magnesium carbonate and bicarbonates, it is believed that these minerals are responsible for the opposite trends in pH observed here. For the solution treated with plasma-microbubbles, pH remains nearly constant after the initial rise in pH despite continuous supply of RNS to the liquid. The minerals contain in tap water may offer mild buffering to the liquid so that the effect of RNS, which typically acidifies water is minimised [23]. It is of interest to note that bubbling alone increases the solution pH faster than that of mixing with an overhead stirrer demonstrating the effectiveness of microbubbles for maintaining well-mixed conditions in the reactor.

A consequence of pH of the bulk liquid remaining above 7 is that it limits the efficiency of the pretreatment. At high pH values, ozone solubility in water is relatively low; hence these conditions will limit mass transfer of O_3_ from microbubbles to the liquid reducing the efficiency of pretreatment [27]. Also basic conditions are less preferential for dissolved ozone as higher pH solutions promote decomposition of ozone through multiple pathways to oxygen [28,29]. Additionally, ozone preferentially attacks the crystalline sections of cellulose under acidic conditions while basic conditions promote attacking both amorphous and crystalline sections [30]. Therefore, to increase the effectiveness of pretreatment, pH manipulation of the liquid phase was explored.

### 3.3 Buffered liquids for pretreatment

**Fig. 6:**
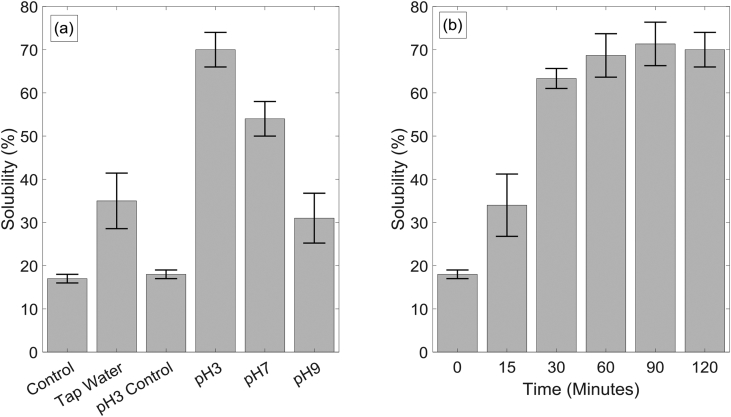
(a) Solubility of α-cellulose treated in plasma-microbubble reactor at different pH buffers and tap water. Two control experiments were run with tap water and pH 3 buffer separately without the plasma effects (b) solubility of α-cellulose in pH 3 buffer with treatment time.

To maintain the pH of the reactor, pretreatment solutions were buffered to pH 3, 7 and 9. The treatment time and the duty cycle were fixed at 2 h and 45% respectively in all cases. Two control tests were run with bubbles, but without the plasma effect: one test with only tap water and the other with a pH 3 buffer. Results are shown in Fig. 6 (a). A significant improvement can be observed for pretreatment carried out with pH 3 buffer, increasing the solubility from 18% to 70%. This increase in cellulose solubility can be attributed to selective oxidation of the crystalline structure at low pH values and to increased dissolved ozone concentration. Henry's constant for bubbly flow ozone transfer increases by 10% changing pH from 9 to 3 at 25 °C [27]. The cellulose solubility observed for pretreatment at pH 7 (54%) is also an improved result from ∼30% solubility reported for both tap water and pH 9 buffer. Fig. 6 (b) shows the effect of treatment time on solubility under the best buffered condition identified, i.e pH 3. Solubility increases rapidly to 63% in the first 30 min of pretreatment at a constant rate followed by plateauing behaviour to reach ∼70% solubility soon afterwards. While this result indicates that pretreatment in the plasma-microbubble reactor is not mass transfer limited, further improvements to solubility require analysing pretreated samples.

Comparison of solubility values achieved in this study with literature is difficult due to the differences in chemicals and conditions used in various studies such as solvent, grade of cellulose, plasma power and process volume. Among the most successful studies reported [14], achieved 95% solubility in sodium hydroxide using a plasma jet after a 90-s treatment at 150 W. Delaux et al. used non-thermal atmospheric plasma (NTAP) to treat MCC with a CI less than 10 and achieved a solubility of 90% in DMSO after a 15-min exposure at 15 W [31]. Although higher solubility values were reported with both studies mentioned above, these are highly energy intensive treatments where cellulose is exposed to plasma directly. Whilst such technologies may be beneficial in processing high value products such as solar cells [32], cost effective and scalable technologies are better suited for low-value high-volume commodities such as biofuels.

**Fig. 7:**
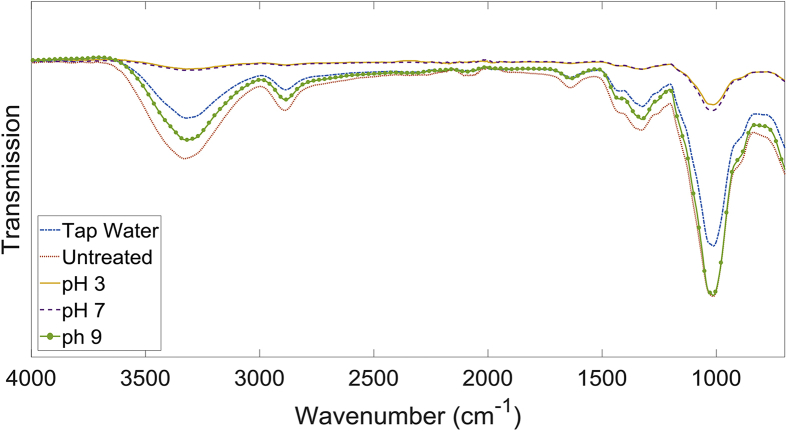
The normalised ATR-FTIR spectrum for samples treated under different buffered conditions.

To investigate structural changes in α-cellulose following pretreatment, ATR-FTIR spectrum was analysed for all pretreated and raw samples. Results are presented in Fig. 7. The spectrum for α-cellulose treated in acidic and neutral buffer solutions overlapped and significantly differ from that of the untreated material. Spectrum for cellulose pre-treated under pH 9 and in tap water also displayed a considerable difference compared to that of untreated cellulose, but the effects were not distinctive as seen under acidic or neutral pH conditions. The decrease in broad peak area observed at ∼3300 cm^−1^ corresponds to the intermolecular and intramolecular O—H bonds within α-cellulose. The reduction of this peak is a key indicator that hydrogen bonds holding the crystalline structure is in fact cleaved by the plasma treatment; hence explains why increased solubility was achieved at low pH conditions [5,14,16]. There is further evidence of decrystallisation of α-cellulose, where a decrease in the peak at ∼1430 cm^−1^ that corresponds to the crystallinity of the structure is observed [33]. Additionally, decrease in the peaks at 2890 cm^−1^ and 1020 cm^−1^, which corresponds to C—H bonds, and 1320 cm^−1^, which corresponds to C—O bonds, show further disintegration of the structure within α-cellulose [34,35]. The increase in solubility reported above is a consequence of cleavage of these hydrogen bonds and change in morphology of the cellulose structure [25].

**Fig. 8:**
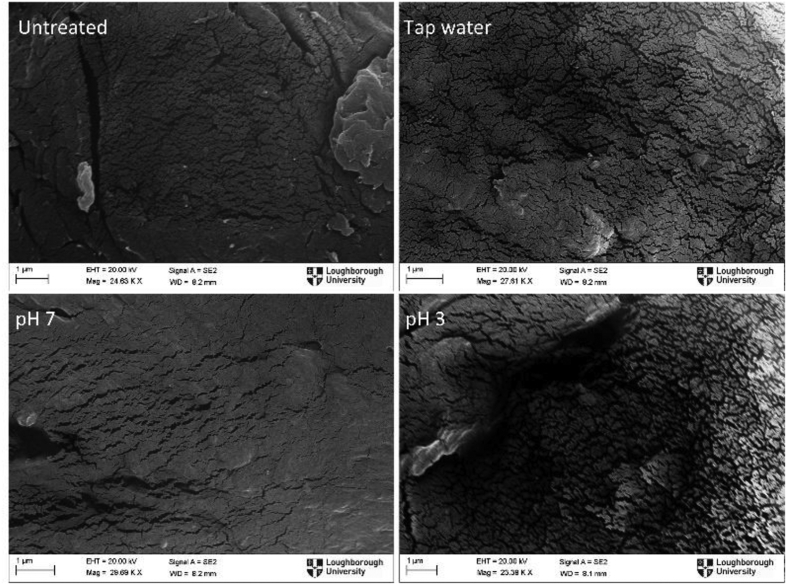
SEM micrographs of raw and pretreated cellulose under different pH conditions.

A qualitative assessment of the pretreated cellulose was carried out using SEM imaging, where changes to the surface of α-cellulose can be observed. Fig. 8 shows micrographs of α-cellulose pretreated under different conditions. The features observed in these micrographs may vary depending on many factors such as cellulose strand observed, shape of the strand and location selected for observation; hence these images should be interpreted with caution. The images presented in Fig. 8 were selected out of many images to represent the general observations seen under each pretreatment condition. Overall, no significant difference is seen between images apart from the sample treated under pH 3. However, careful observation suggests that small crevices found in untreated (raw) cellulose swells during all pretreatment conditions in addition to creating more cracks on the surface. In some cases, these crevices merge together to form more significant cracks on the surface as in the sample treated under pH 3. This break-up of the surface structure facilitates increased transfer of reactive species beyond the top surface and allows bond cleavage deeper within the structure. However, SEM micrographs cannot discriminate between the amorphous and crystalline sections oxidised by O_3_.

The main purpose of biomass pretreatment is to prepare the material for hydrolysis and subsequent fermentation or digestion by bacteria; hence not only the surface but the entire biomass particle should open-up providing access to cellulose and hemicellulose. It is common practice to reduce the particle size by milling to increase the surface area and to reduce the distance reactive species should travel by diffusion to reach the centre of the particles [36]. Whilst the SEM images in Fig. 8 show an increased break-up of the surface providing more routes for attack on cellulose fibres, transfer of reactive species to the internal structure of strands is mainly by diffusion in the narrow crevices. Assuming no convection and no reactions with the walls, it is possible to calculate the time it takes for an ozone molecule to reach the centre of a strand from its surface as 0.045 s, considering a strand diameter of 25 μm estimated from SEM images and using a diffusion coefficient of $1.75\;\times 10^{-9}$ m^2^s^−1^ [37]. This estimated time is much smaller than the treatment time of 2 h. However, when the crevices are only few nanometres wide as in this case, interaction between the polar ozone molecules and the internal walls dominates the mass transfer process. The surface charge on the internal walls within the crevices is governed by the solution pH and the ions present in the solution as a result of the dissolved plasma products. Additionally, ozone reacts with the side walls within the crevices and the actual time to treat surfaces within the cracks could be much longer than the estimated time. Based on the above analysis, it is hypothesised that the high rate of solubility increase observed for the first 30 min (see Fig. 6(b)) relates to the treatment of exposed particle surfaces while the plateauing behaviour of the solubility curve is related to the diffusion limited mass transfer within crevices. A typical approach to increase diffusion dominated transport is to increase temperature of the liquid; however, this will adversely affect mass transfer rates from bubbles to liquid and will also increase decomposition of dissolved ozone.

### 3.4 Repeated batch processing

**Fig. 9:**
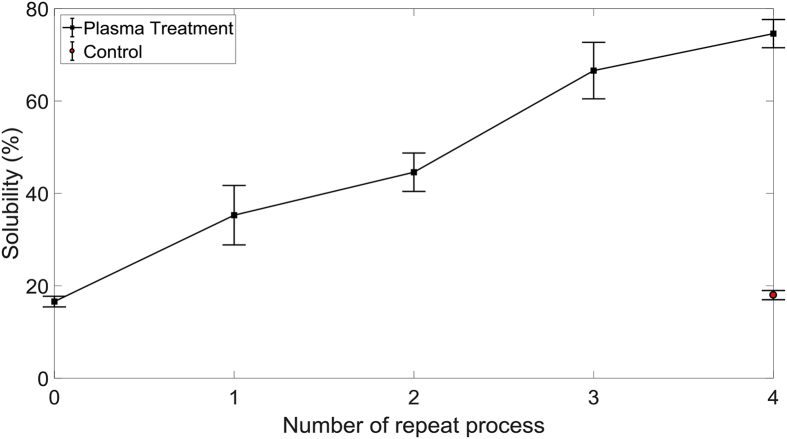
The effect of repeated treatments on α-cellulose. Each cycle is pretreated for 2 h followed by filtration, washing, drying and resuspension in fresh tap water before the next cycle of treatment. Error bars represent standard error.

Whilst the use of buffers allows increased breakup of α-cellulose, there are clear drawbacks such as the need for additional chemicals and chemical recovery post-treatment, increasing the cost of the overall process. With the aim of overcoming the need for additional chemicals and to increase the solubility of α-cellulose, samples were treated as a batch several times. After each 2-h pretreatment, cellulose was separated from the liquid by filtration, washed and dried before subjected to further treatment with fresh tap water. This method of repeated batch processing of cellulose was found to increase the solubility considerably, as shown in Fig. 9. Each processing cycle improved the solubility by a similar fraction, and after the sample was treated 4 times, a cumulative solubility of ∼75% was achieved. The control experiment, where α-cellulose was bubble treated without plasma for 4 cycles and washed at the end of each cycle, showed no changes to solubility. It was speculated that any by-products generated during the pretreatment process is hindering further cleavage of the cellulose structure [38], but removal of the liquid and resuspension seems to have solved this issue temporarily until the subsequent treatment cycle matures. Interestingly, this was not the case for buffered treatment under pH 3. It is possible that any interference from the by-products formed during pretreatment and their interaction with the cellulose structure is minimal at acidic pH. Also, it is possible that this inhibitory product is either not formed or undergoes a chemical reaction at acidic conditions. Whilst this method offers an alternative to buffered-pretreatment with a reduced chemical demand, this approach will increase the power consumption, quantity of water used and processing costs.

Whilst treatment with APP has shown to depolymerise cellulose structure through cleavage of the hydrogen bonds, random repolymerisation reactions have also been reported [39]. Delaux et al. found that mono- and disaccharides subjected to DBD plasma starts to polymerise when the reactor temperature reaches 40 °C [40]. DBD plasma is a source of heat and the liquid temperature could rise gradually exceeding 40 °C locally near the membrane leading to repolymerisation. While this may explain the plateauing behaviour seen in Fig. 4, Fig. 6 for continuous treatments, halting the treatment process and resuspending cellulose in fresh tap water lowers the suspension temperature promoting depolymerisation reactions to dominate once again.

### 3.5 Glucose conversion efficiency

**Fig. 10:**
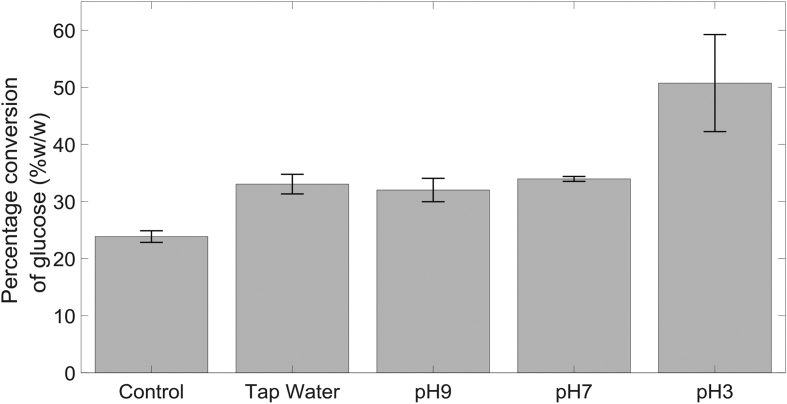
Conversion of α-cellulose to glucose after pretreatment under different buffered conditions. Error bars represent standard error.

This work has demonstrated effective break-up of crystalline structure of α-cellulose; however, it is important to demonstrate that the process does not produce inhibitors for the subsequent step: hydrolysis. Therefore, enzymatic hydrolysis was carried out on the pretreated samples under buffered conditions to convert α-cellulose into glucose. Saccharification results are shown in Fig. 10. For tap water and buffered liquids at pH 7 and pH 9, pretreatment increased the glucose conversion from ∼24% to ∼33%. Apparently, changes to the solubility observed in Fig. 6 (a) for these suspensions are not reflected in the glucose conversion results. Enzymes are highly specific to functional groups or bonds in the cellulose structure; hence any changes to the cellulose structure as a result of pretreatment could affect the glucose yields [41,42]. However, suspensions pretreated under pH 3 buffer increased glucose conversion to ∼51%. For the samples pretreated under pH 3 buffer, crevices observed with SEM micrographs were larger and more pronounced. This would suggest that the cracks propagated on the surface of cellulose were essential in achieving high conversion yields, and the main limiting factor is the accessibility of species/enzymes to the internal surfaces of the cellulose stands.

A higher glucose conversion efficiency was reported by Ref. [43], where α-cellulose was dissolved in NaOH/urea solution as a pretreatment step to disrupt the crystalline structure before saccharification. Cellulose precipitated from NaOH/urea solution were separated and subjected to hydrolysis for 3 days, which achieved 96% conversion. However, this approach requires the use of highly concentrated chemicals: 2 M sodium hydroxide and 1.75 M urea. This is in stark comparison to the pH 3 buffer solution used in this study with a concentration of 0.1 M. Even though plasma-microbubble pretreatment at current state may not seem competitive compared to established chemical techniques, it has a great potential to reduce chemical use in the pretreatment step. To increase the conversion yield of α-cellulose to glucose with the plasma-microbubble pretreatment presented in this paper, higher concentrations of ROS in the liquid phase could be beneficial. It is possible to increase generation of ROS by providing additional cooling of the plasma module combined with operating at higher duty cycles, but dispersal of the generated species in the liquid should not be overlooked. Gas bubbles on the order of 500 μm were produced in this study using a hydrophilic microporous membrane. However, if combined with a fluidic oscillator, these membranes can produce monodisperse microbubbles ∼100 μm and improve gas-liquid mass transfer rate significantly [44]. The power consumption of the plasma increases linearly with the duty cycle used, and for a 45% duty cycle, the reactor draws 19 W of power. Therefore, the power consumption of cellulose to glucose conversion under the best operating conditions is estimated to be 3.8 kW h/kg of cellulose. Pulidindi et al. used microwave irradiation, another widely used approach for hydrolysis, to convert commercial cellulose to glucose which consumed 149 kW/kg of cellulose [39]. Clearly, power consumption for plasma-microbubble treatment investigated in this study is significantly less than that of microwave irradiation reported in the literature demonstrating the energy efficiency of the process. Further work is required to assess the feasibility of this approach as a potential pretreatment method for reducing cellulose recalcitrance to hydrolysis.

## 4 Conclusions

α-cellulose, a renewable polysaccharide with a highly ordered crystalline structure, was pretreated using a plasma-microbubble reactor, where the plasma discharge was formed in the vicinity of the microbubble generation sites. Pretreatment carried out with suspensions buffered at pH 3 provided the best conditions increasing cellulose solubility from 17% to 70%, when treated for 2 h. The rate of pretreatment was found to be high for the initial 30-min period followed by plateauing behaviour afterwards, suggesting a limitation to further treatment. Alternatively, repeated batch processing with resuspension in fresh water increased solubility to ∼75% after 4 cycles. The effectiveness of the pretreatment was also assessed using ATR-FTIR, where evidence was apparent that the hydrogen bonds linking the polymeric structure were cleaved successfully. Furthermore, SEM micrographs showed pronounced crevices at the cellulose surface following pretreatment under best conditions. Breaking up of the polymeric structure of α-cellulose also translated into higher glucose conversions by enzymatic hydrolysis. As with the solubility study, pH 3 buffered solutions provided the most favourable conditions for saccharification increasing the conversion efficiency from 24% to 51% after 7 days.

The findings of this study reaffirm the barriers to pretreatment and saccharification found by previous authors: i.e limited accessibility of reactive species and enzymes to the crystalline cellulose structure. Also, our study confirmed that the dissolved reactive species produced by the plasma were able to form significant crevices under suitable conditions; hence providing a partial solution to the accessibility issue. This study raises the question whether higher doses of O_3_/reactive species would suffice utilizing major proportion of cellulose for sugar conversion. Clearly, this study demonstrates a promising approach for pretreatment with a reduced chemical demand, but further work is required to increase and match saccharification efficiencies achieved with well-established high chemical demand dissolution treatments.
